# Intelligence

**Published:** 1826-08

**Authors:** 


### INTELLIGENCE.

### MONTHLY REPORT OF PREVALENT DISEASES.

During the past month, we have experienced more than the usual vicissitudes of our uncertain climate: the thermometer has stood so high as 88°, and so low as 52°; while we have had calms and heavy gales of wind,—many successive days of a burning sun and cloudless sky, and others of continued rain. These changes, however, have produced neither so great a variety of diseases, nor so many invalids, as might have been expected. The leading ailment has been derangement of the digestive system: this has frequently been attended with a quick, small pulse, a yellow tongue, diarrhœa, and considerable debility; but in scarcely any instance has it assumed the character of true cholera. These cases have become less numerous since the weather became cooler, and pulmonary diseases have increased proportionally.—Rheumatism was decidedly more prevalent during the very hot, than during the cooler period of the month.—In our last report we mentioned the case of a gentleman who had recovered from an apoplectic seizure; he has since perished from a return of the disease: no morbid appearance whatever was found in the brain. We have seen two other apoplectic affections, neither of which have proved fatal: of these we may, perhaps, give a more detailed account on a future occasion.

Measles has prevailed to a considerable extent in some districts: we lately ascertained the existence of thirteen cases in a court containing only twelve houses.

---

### *Note respecting the Case of James White, published in our last Number.*

This, it will be remembered, was a case in which a tobacco glyster had been administered, prior to the patient's admission into the Middlesex Hospital, and in which the scrotum was discoloured, apparently from having been bruised. The following note is from Mr. Tweedale:

"In your last Number, Mr. Shaw has published a case of Hernia, and attributed the symptoms to the effects of a tobacco-glyster, given before the man arrived at the hospital. The following statement may, perhaps, put the case in a different point of view:—About four o'clock in the morning of the 9th of December, 1825, James White, a patrol, while in pursuit of a thief, ran violently against a post, and received a severe blow on the abdomen. A large scrotal hernia immediately came down. Two hours afterwards, he was brought to St. Giles's Infirmary. He then complained of severe pain of his belly, and vomited frequently; his countenance was sunk, and indicative of great distress; and the pulse nearly imperceptible. On being put to bed, he rallied a little, but the vomiting and pain continued. A tobacco glyster was given, and the taxis used, but without success. Mr. Ogle (under whose care the man was) then sent him to Middlesex Hospital. As the symptoms referred by Mr. Shaw to the glyster were present before that was given, we must look to some other quarter for their cause. If I may be allowed to hazard a conjecture, I should be disposed to attribute them partly to the shock of a severe blow on the abdomen, and partly to the descent of a considerable portion of intestine. Whatever blackness of the integuments Mr. S. noticed, must be referred to a long-continued application of the taxis at the hospital, before Mr. S. saw the patient; for certainly nothing of the kind was visible when the man left the infirmary."

On the receipt of the above, we wrote to Mr. Shaw, requesting to know whether the history of the case had been correctly given, so far as regarded the period during which the patient was in the hospital. We subjoin his reply:

"Whatever may have been the patient's condition before the tobacco glyster was administered, there can be little doubt that the extraordinary depression he laboured under, for the first ten hours he was in the hospital, was caused principally by the tobacco; for the symptoms were not only exactly similar to those presented in cases where this remedy has been used, but the patient recovered gradually, and in the manner a person rallies after having been sick from smoking or chewing tobacco. I shall, perhaps, on another occasion, offer you some remarks on the soundness of the theory on which the practice of using the tobacco glyster is founded, and on the obscurity which it frequently produces in the nature of the symptoms. But I cannot at present avoid expressing my surprise, that your correspondent should have administered the tobacco when the patient was in the state he has described, as the most strenuous advocates for its employment advise it only as the means of producing that condition under which it would appear that this patient already laboured. With regard to the bruised condition of the scrotum, as White acknowledged having made use of violent efforts to reduce the rupture, this appearance may fairly be attributed to his own unskilful surgery, without blaming his medical attendants, either before or after his admission into the Middlesex Hospital."

---

### *On the Qualifications of Medical Officers in the Army.*

*Hospital Assistant.*—The candidate for this appointment in the medical department must be unmarried, not under twenty-one nor above twenty-six years of age; he must produce certificates of having served a regular apprenticeship, of not less than three years, (to a member of either of the Royal Colleges of Surgeons of a chimney-sweep. Serves eight years

London, Dublin, or Edinburgh, will be preferred,) and of attendance, for one year at least, at an hospital or infirmary of celebrity. Without apprenticeship, two years' attendance of an hospital, with one on practical pharmacy, will be required. He is to possess a diploma from one of the Royal Colleges mentioned, and must exhibit certificates of regular courses of study in the following branches of professional knowledge, at established schools of eminence, viz.—

| | |
|---|---|
| Anatomy | 18 months, |
| Practical Anatomy | 12 ditto, |
| Chemistry | 6 ditto, |
| Materia Medica | 3 ditto, |
| Surgery | 6 ditto, |
| Theory of Medicine and Physiology | 6 ditto, |
| Practice of Medicine | 6 ditto, |
| Botany | 6 ditto, |

And of Clinical Lectures on the Practice of Medicine and Surgery.

It will be considered an additional recommendation to gentlemen entering the service, to have attended Lectures on Forensic Medicine, and public establishments for the treatment of diseases of the Eye and Skin, and of Mental Derangement.

Gentlemen who have had an university education will be preferred. It is desirable that all should have studied natural philosophy, mathematics, and natural history in all its branches; but a liberal education, and a competent knowledge of the Greek and Latin languages, are indispensably requisite in every candidate.

The greater the attainments of the candidates in various branches of science, in addition to competent professional knowledge, the more eligible will they be deemed for promotion; for selections to fill vacancies will be guided more by reference to such acquirements, than to mere seniority. With the above-recited qualifications, officers are entitled to promotion as assistant surgeons and regimental surgeons; but every gentleman must have served five years at least in the junior appointments, before he can be promoted to the rank of regimental surgeon; and he who gives the best proofs of diligent exertion in the performance of public duty, and of attention in the acquirement of practical knowledge, will be noted as the most eligible candidate for advancement.

Gentlemen already in the service are earnestly recommended to avail themselves of every opportunity of adding to their knowledge, by attending universities or schools; for which purpose, every facility in his power will be afforded by the Director General. They are especially desired to transmit to this office, the statements of such classes as they may have attended subsequently to their examinations before this Board, either on professional or other branches of science, that the same may be duly registered: and every gentleman must be prepared for further examination, before he can obtain promotion, or return to the service from the half-pay list.

Medical officers are encouraged and recommended to look forward to the appointment of Surgeon to the Forces, and of Physician to the Forces; and to endeavour especially to qualify themselves for either, according to the bent of their inclinations, and to their previous study.

For the commission of Surgeon to the Forces, it is expected that the candidate shall have attended a public hospital of celebrity at least two years, and it is desirable that one of them should have been passed at a London hospital.

The rank of Physician to the Forces requires, in addition to the knowledge and experience to be gained in the regular progress of study and of service, that the candidate should be a Fellow or Licentiate of the Royal College of Physicians of London, or a graduate of the university of Oxford, Cambridge, Dublin, Edinburgh, Glasgow, or Aberdeen.

Although the British schools are specified, it is to be understood that candidates, who have received regular education in approved foreign universities or schools, will be admitted to examination.

J. M'GRIGOR, *Director General.*
W. FRANKLIN, *Principal Inspector.*

*Army Medical Department; 1st July,* 1826.

The following has been transmitted to us for insertion by the Secretary to the Association.

### *Report of the Associated General Medical and Surgical Practitioners.*

The anniversary meeting of the Associated General Medical and Surgical Practitioners, hitherto known under the title of the "Associated Apothecaries and Surgeon-Apothecaries of England and Wales," was held at the Crown and Anchor Tavern, in the early part of last month; and the following Report presented:—

Faithful to the trust reposed in them, your committee respectfully submit to the general meeting a report of their proceedings during the past year.

From the passing of the Act of 1815, commonly called "the Apothecaries' Act," the committees, successively appointed by the Association, have been anxious to extend the salutary provisions of that law, and to remedy its glaring defects; as well as to increase, through more efficient examinations, the usefulness of the practitioner, and the respectability of the profession to which he belongs.

Early in the last session, your committee determined on renewing their application to Parliament; and, after a conference of the deputation (formerly appointed) with Mr. Hume, they resolved to present a petition on the subject of those complaints, which the Apothecaries' Society continued to overlook, or affect to despise, even while (under the dread of losing their cherished Act altogether) they were remodelling their Court of Examiners, and improving their methods of examining candidates.

Meanwhile, the conduct of the Council and Court of Examiners of the College of Surgeons having excited much animadversion, and produced a disposition generally on the part of the profession to remonstrate on the impropriety, and even the injustice, of certain newly adopted regulations of theirs, it occurred to your committee, that, as persons entrusted by a numerous and highly respectable portion of the professional public with its welfare, it was particularly incumbent on them to take the necessary steps for showing their sense of the extraordinary conduct of the College; to assist in redressing the evils which were likely to arise, both to the community and to the profession, from such conduct as had recently been displayed; and especially to put the practice of Midwifery into such a state as would better secure the safety of human life.

That the deliberations contemplated might have all the aid which could be derived from numbers, experience, and talent, it was resolved to call a meeting,—not merely of the members of this Association, which might appear much too limited for the inquiry, but of general practitioners, who might represent fully the feelings and wishes of the majority of practitioners, resident in or near the metropolis; and, further, under these circumstances, your committee deemed it fair to defray, out of the funds of the Association, all the expences which should accrue from convening such a meeting; but, once assembled, it would remain for that meeting, out of its own resources, to adopt and prosecute such remedial measures as might seem to them best suited to the occasion.

Your Committee believed that, in thus acting, they were, without departing from the tenor of their injunctions, performing an important public duty; and that the great body of general practitioners would gladly avail themselves of the opportunity thus afforded to express the sentiments they entertained on very various subjects connected with the profession, and to concur in such measures as might appear just and necessary.

The meeting, which was held on the 25th of February last, (a great majority of which were members of the College of Surgeons,) abundantly confirmed the propriety of the reasoning of your committee: it was very numerously and most respectably attended; and the Resolutions then proposed, and at a subsequent meeting confirmed, became the foundation of a petition to the House of Commons, which, after specifying the existing grievances, prayed the honourable House to institute an inquiry into the present state of medicine throughout England and Wales,—to cause efficient examinations as to the qualifications of all persons about to practise medicine or midwifery,—and to adopt such other measures as, in the judgment of the honourable House, might seem necessary for the remedying of the alleged abuses.

This petition, numerously signed, was presented to the House of Commons by Mr. Hume, on the 9th of April, and was ordered to lie on the table. That no discussion was entered into on its merits, was undoubtedly owing to the extreme pressure of public business,—the shortness of the session,—and to the meditated dissolution of Parliament, which has since taken place.

Various letters from eminent practitioners in the country, expressive of their concurrence in the measures adopted at those meetings, and the subscription cheerfully entered into to defray the expences incurred, are satisfactory testimonials that the course resolved upon was very generally approved; while the whole tenor of the Resolutions passed at these two meetings demonstrate the disinterested conduct and liberal views of the gentlemen who took the lead on these occasions.

Thus, without the smallest deviation from the principles of the Association, your committee have zealously endeavoured to ascertain the sentiments and wishes of a still more extensive class of the profession, and induced them to apply to Parliament for the removal of evils, which can be obviated only by legislative enactment,—sensible that they could not possibly obtain for the public on the one hand, and for the profession on the other, a greater boon than a free and comprehensive inquiry into the present state of medicine, the abuses in which are continually extending themselves, and calling with a louder voice for investigation and redress.

If there were any persons who entertained a hope that, on a future application to Parliament, the Apothecaries' Society would so far consider the feelings of practitioners, and the dignity of the profession, as to withdraw the clauses in their Act, so often and so indignantly complained of, they must now be convinced that such a hope can no longer be reasonably entertained. Within these two years, the Society have been twice before the legislature; and it is painful to observe, that the maintenance of the Act of 1815, with nearly all its original imperfections, and teeming with degradations, has seemed of more value in their eyes, than the respect of their medical brethren, or even the honour of the profession itself.

Your committee, therefore, earnestly recommend to their successors, collectively and individually, to use their utmost endeavours to obtain and diffuse information as extensively as possible; and, immediately after the meeting of Parliament, to present petitions to both Houses, of similar import to those already submitted; not doubting that, as no power can render error perpetual, an enlightened Legislature will sooner or later perceive and remedy all the grievances complained of, and place on its proper basis a profession so highly important to the public good.

The expenditure has been kept within as narrow limits as possible; and the committee confidently trust that, in this as in all other instances, the members of the Association will recognise an earnest desire on the part of the committee to render their labours subservient to the welfare of the community, and to the respectability and usefulness of the general practitioner.

---

### *Instrument for ascertaining the Presence of Animal Matter in the Atmosphere.*

Dr. Granville has invented an instrument, which, by means of a preparation of chlorine, enables him to ascertain, not only whether animal matter, in a state of decomposition, be floating in the air, but also the quantity of such animal matter; a knowledge which we cannot attain by the usual apparatus for analysing atmospheric air, and the importance of which to the medical profession must be obvious. He proposes calling the instrument the *Septometer.*

---

### *Cupping.*

Mr. Kennedy, of Virginia Terrace, has invented a cupping-glass, calculated to facilitate the operation in the hands of the inexperienced. The difficulty with beginners is to produce a sufficient vacuum without the air being too much exhausted, as this causes so much pressure on the skin as to interrupt the circulation through the minute vessels. Mr. Kennedy's contrivance is in principle the same as that we formerly mentioned as adopted by Mr. Clarke. It consists in placing within the glass a small piece of sponge, immersed in spirits of wine; but this, instead of being attached to a bent silver spring, is introduced through an aperture at the fundus of the cup, which is rendered air-tight by a screw armed with a piece of leather. Those unaccustomed to the operation will succeed better with either of these contrivances, than with the common apparatus: those, again, who are accustomed to it will only find themselves encumbered by adopting them.

---

### *New Editions.*

There is considerable justice in the remarks contained in the following note, which we have received from a "Member of the College of Surgeons."

"A very great benefit would be conferred upon the profession, if authors, when called upon for new editions of their works, would publish any alteration of sentiment, or any addition of matter, separately in the form of an appendix. Whereas, in the usual mode of sending forth a new edition of a work into the world, former editions become of comparatively little value, and consequently the property of individuals seriously injured. The mischief does not rest here. Proprietors of early editions have, if anxious to possess themselves of the author's latest sentiments, to make a second purchase. This is a gross tax, and loudly calls for the sentiments of the profession. I would be very far from imputing dishonourable motives to authors who are almost yearly invading the property of their brethren. There can be no doubt but that, on their part, the injury is quite unintentional; and let it be pointed out to them through the aid of your Journal, and I trust that, in a short time, we shall have the satisfaction to find improvements and alterations published separately, at comparatively trifling expense."

### METEOROLOGICAL JOURNAL,

*From June 20th, to July 20th,* 1826.

By Messrs. HARRIS and Co. Mathematical Instrument Makers, 50, High Holborn.

| June | Moon. | Rain gauge | Thermom. | | | Barometer. | | De Luc's Hygrom. | | Winds. | | Atmospheric Variations. | | |
|---|---|---|---|---|---|---|---|---|---|---|---|---|---|---|
| | | | 9 A.M. | MAX. | MIN. | 9 A.M. | 10P.M. | 9 A M. | 10P.M. | 9 A.M. | 10 P.M. | 9 *a.m.* | 2 *p.m.* | 10 *p.m.* |
| 20 | | | 64 | 71 | 52 | 30.30 | 80.30 | 61 | 66 | ENE | ESE | Fine | Fair | Fair |
| 21 | | | 61 | 67 | 53 | 29.25 | 29.24 | 65 | 74 | NNE | NE | Cloudy | — | Sm. Ra. |
| 22 | | | 66 | 75 | 52 | 30.20 | 30.21 | 72 | 72 | NE v. | E | Fair | Fine | Fair |
| 23 | | | 57 | 73 | 52 | 30.24 | 30.25 | 79 | 59 | NE | NE | — | — | — |
| 24 | | | 65 | 78 | 57 | 30.25 | 30.22 | 61 | 63 | ENE | ENE | Fine | — | — |
| 25 | | | 73 | 79 | 57 | 30.17 | 30.13 | 53 | 61 | ESE | E | — | — | Fine |
| 26 | | | 67 | 83 | 65 | 30.08 | 30.00 | 61 | 61 | E | E | — | — | — |
| 27 | | | 70 | 86 | 70 | 29.86 | 29.89 | 62 | 58 | NNW | S | — | — | Cloudy |
| 28 | ☾ | | 75 | 88 | 65 | 29.92 | 29.99 | 61 | 62 | SW v. | SW | — | Sm.Sho. | Fair |
| 29 | | | 76 | 81 | 64 | 30.03 | 30.06 | 63 | 72 | W | W | — | Fine | — |
| 30 | | .6 | 70 | 82 | 66 | 30.06 | 80.03 | 66 | 61 | WSW | W | Cloudy | — | Rain |
| July | | | | | | | | | | | | | | |
| 1 | | | 69 | 78 | 82 | 30.04 | 30.05 | 70 | 68 | SW | SW | — | — | Fair |
| 2 | | | 76 | 80 | 65 | 30 09 | 30.12 | 61 | 58 | WNW | SW | Fine | — | Fine |
| 3 | | | 75 | 83 | 63 | 30.10 | 30.05 | 62 | 63 | SE | E | — | — | — |
| 4 | | | 70 | 82 | 67 | 29.95 | 29.84 | 63 | 69 | SSE | E | — | — | — |
| 5 | ● | | 80 | 82 | 65 | 29.75 | 29.77 | 60 | 64 | SSW | SW | — | — | Cloudy |
| 6 | | | 73 | 81 | 68 | 29.80 | 29.75 | 70 | 63 | W | WSW | Cloudy | — | Fine |
| 7 | | | 79 | 82 | 65 | 29.69 | 29.64 | 65 | 66 | W | SSW | Fine | — | — |
| 8 | | .8 | 74 | 81 | 67 | 29.58 | 29.58 | 65 | 73 | S | WSW | — | — | Rain |
| 9 | | | 75 | 80 | 64 | 29.57 | 29.60 | 69 | 60 | SSW | W | Fair | — | Fine |
| 10 | | | 73 | 78 | 61 | 29.65 | 29 72 | 61 | 61 | WSW | W | — | — | Fair |
| 11 | | | 65 | 75 | 62 | 29.74 | 29.81 | 63 | 63 | WSW | WNW | — | — | — |
| 12 | ☽ | | 65 | 76 | 65 | 29.76 | 29.66 | 70 | 72 | WNW | SW | Sm. Ra. | Fair | Fine |
| 13 | | .11 | 70 | 72 | 59 | 29.55 | 29.55 | 72 | 83 | WSW | SW | Fair | Rain | — |
| 14 | | | 65 | 75 | 52 | 29.62 | 29.72 | 72 | 60 | W | SW | — | Fair | — — |
| 15 | | | 69 | 74 | 59 | 29.74 | 29.75 | 65 | 61 | SW | WSW | Fine | — | Rain |
| 16 | | .5 | 65 | 70 | 55 | 29.66 | 29.75 | 75 | 62 | SW | WNW | Rain | — | Fair |
| 17 | | | 65 | 73 | 59 | 29.83 | 29.83 | 62 | 63 | N | WSW | Fine | — | — |
| 18 | | | 70 | 73 | 61 | 29.82 | 29.78 | 59 | 63 | WNW | W | — | — | — |
| 19 | | | 67 | 73 | 59 | 29.78 | 29.90 | 65 | 58 | WNW | WNW | — | — | — |

The quantity of Rain fallen in the month of June, was 62.100ths of an inch.

